# Evaluation of the Choroid Plexus Epithelium Inflammation TLR4/NF‐κB/NKCC1 Signal Pathway Activation in the Development of Hydrocephalus

**DOI:** 10.1111/cns.70085

**Published:** 2024-10-25

**Authors:** Hao Xu, Jiawei He, Hua Du, Xiaolei Jing, Xinfeng Liu

**Affiliations:** ^1^ Department of Neurosurgery, The First Affiliated Hospital of USTC, Division of Life Sciences and Medicine University of Science and Technology of China Hefei Anhui China; ^2^ Department of Neurosurgery, The First Affiliated Hospital of Xiamen University School of Medicine, Xiamen University Xiamen Fujian China; ^3^ Key Laboratory of High Magnetic Field and Ion Beam Physical Biology Hefei Institutes of Physical Science, CAS Hefei Anhui P. R. China; ^4^ Anhui Province Key Laboratory of Environmental Toxicology and Pollution Control Technology Hefei Institutes of Physical Science, CAS Hefei Anhui P. R. China; ^5^ Department of Neurology, The First Affiliated Hospital of USTC, Division of Life Sciences and Medicine University of Science and Technology of China Hefei Anhui China

**Keywords:** AQP1, choroid plexus epithelium, hydrocephalus, inflammation, NKCC1

## Abstract

**Background:**

Hydrocephalus is characterized by secretion, circulation, and absorption disorder of cerebrospinal fluid (CSF) with high morbidity and complication rate. The relationship between inflammation and abnormal secretion of CSF by choroid plexus epithelium (CPE) had received more attention. In this study, we aim to detect the role of Toll‐like receptor 4/nuclear factor‐kappa B/Na+/K+/2Cl‐cotransporter 1(TLR4/NF‐κB/NKCC1) signal pathway in the development of hydrocephalus.

**Method:**

Hydrocephalus was induced in adult rats (8 weeks) by intracisternal kaolin injection, then pyrrolidinedithiocarbamate (PDTC) and bumetanide were administrated to the rats mode. Then the rat model was evaluated, and ventricular volume was calculated at different time points. Then CPE, cortex, preventricular tissue, and CSF were obtained. Protein expressions of TLR‐4, NKCC/serine–threonine STE20/SPS1‐related, proline‐alanine‐rich kinase (SPAK), pNKCC1, pSPAK, GFAP, AQP1, and AQP4 were measured by RT‐PCR, western blot, and immunofluorescence (IF) stains in CPE, respectively.

**Result:**

Our data showed that inflammation factors tumor necrosis factor‐(TNF‐α), interleukin 18(IL‐18), and glial fibrillary acidic protein (GFAP) concentrations were significantly higher in the model group than in controls. The TLR4/NF‐κB/NKCC1 signal pathway were actived by NF‐κB‐p65, NKCC1, pNKCC1‐ pSPAK complex, and Aquaporin1 (AQP1) high expression. PDTC and bumetanide use can help regular TLR4/NF‐κB/NKCC1 expression and reduced AQP1 expression by down‐regulate NF‐B‐p65 and inhibiting NKCC1, respectively. As a result, the treatment groups alleviated CPE abnormal secretion and ventricle enlargement.

**Conclusion:**

These results confirmed that the inflammatory reaction contributes TLR4/NF‐κB/NKCC1 mediated CPE abnormal secretion and consequent hydrocephalus. Regulation of TLR4/NF‐κB/NKCC1 and AQP1 can prevent this process. Our study provides a strong rationale for further exploring alleviating CPE abnormal secretion as a therapeutic perspective of hydrocephalus.

## Graphical abstract

CPE abnormal secretion could be a therapeutic perspective of hydrocephalus. TLR4/NF‐κB/NKCC1 and AQP1 can prevent the prosses of hydrocephalus. Inhibit TLR4/NF‐κB‐dependent inflammation might avoid permanent shunt dependence.

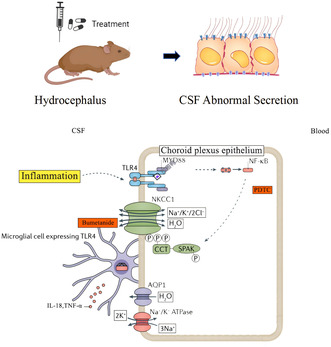

**Abbreviations**
AQP: aquaporin
CPE: choroid plexus epithelium
CSF: cerebrospinal fluid
IF: immunofluorescence
IL‐18: interleukin 18
NF‐κB: nuclear factor‐kappa B
NKCC1: Na+/K+/2Cl − cotransporter 1
PDTC: pyrrolidine dithiocarbamate
SPAK: serine–threonine STE20/SPS1‐related, proline‐alanine‐rich kinase
TLR4: toll‐like receptor 4
TNF‐α: tumor necrosis factor‐α
TNF‐α: tumor necrosis factor‐α

## 1 Introduction

Hydrocephalus is a common medical condition characterized by abnormalities in the secretion, circulation and resorption of cerebrospinal fluid (CSF), resulting in ventricular dilatation [1]. However, the mechanisms underlying these deficits are not fully understood. Previous studies have confirmed that inflammation plays an important role in the development of hydrocephalus and affects the entire CSF absorption system. In the early and middle stages of hydrocephalus, the relationship between inflammation and abnormal secretion of CSF by choroid plexus epithelium (CPE) received more attention recently [2, 3].

Initially, this secretory response may be adaptive in maintaining homeostasis by clearing pathogenic organisms or debris from the epithelial surface [4, 5]. Hyper‐secretion of CSF due to CPE hyperplasia or choroid plexus tumors is sufficient to cause non‐obstructive hydrocephalus [6, 7]. Although endoscopic cauterization or the removal of the CPE has proven effective in some hydrocephalus patients, the surgical risk and the influence of some endocrine function can not be ignored. So that it can not be widely used [8, 9, 10]. In contrast, acute delivery of repurposed drugs targeting either Toll‐like receptor 4/nuclear translocation of nuclear factor‐κB (TLR4/NF‐κB)‐dependent inflammation or the serine–threonine STE20/SPS1‐related, proline–alanine‐rich kinase/Na+/K+/2Cl − cotransporter 1 (SPAK‐NKCC1) complex might serve as a novel strategy to avoid permanent shunt dependence while preserving other critical CPE functions [11, 12, 13, 14, 15].

A recent rat model demonstrates how blood in the cerebral ventricles triggers TLR4‐mediated CSF hypersecretion by the CPE [4]. These results suggest that in addition to microglia, MyD88‐dependent TLR4 signaling may play a critical role as an inflammatory mediator on other CNS cell types as well [16, 17]. Aquaporin (AQP) is a recently discovered protein regulating fluid homeostasis in various organs [4, 18, 19]. AQPs can transport water bi‐directionally across cell membranes in response to changes in passive osmotic pressure gradients as they have a high capacity and more excellent selectivity for the water molecules. AQP1 is mainly expressed in the apical membrane of choroid plexus epithelial cells, which suggests a role in CSF secretion [20, 21].

Therefore, we established a non‐obstructive hydrocephalus rats model by kaolin inducing and elucidating water physiology in hydrocephalus and inflammation level. Then regulating nuclear factor‐κB (NF‐κb) mediated pNKCC1‐pSPAK complex in choroid plexus epithelial to evaluate the influence of AQPs and CSF metabolism. This study was designed to investigate AQP1 expression in specific brain regions corresponding to the severity of hydrocephalus, while detecting whether regulating the NF‐κb mediated pNKCC1‐pSPAK complex in choroid plexus epithelia can ease the process of hydrocephalus.

## 2 Method

### 2.1 Animals

Adult male Sprague–Dawley rats (240–260 g) were housed in a temperature‐controlled room under specific pathogen‐free conditions and a standard 12‐h light/dark cycle, with ad libitum access to food and water. Then the rats were randomly divided into four groups: experimental group (*n* = 35) with kaolin injections, sham operation group (*n* = 20), PDTC group (*n* = 15) and Bumetanide group (*n* = 15) with saline injections. The number of rats killed at various points in different groups is shown in Table 1. All procedures were performed according to the Institutional Animal Care and Use Committee guidelines of USTC (2021NA‐011). Animals in this study were randomly chosen for either control or experimental conditions, the researchers were not blinded, and no animals were excluded.

**TABLE 1:** Number of rats killed at various points in different groups.

	0 day	1 day	3 days	7 days	14 days
Sham control group		5		5	5
HA group	5	5	5	5	5
PDTC group		5	5	5	5
Bumetanide group		5	5	5	5

### 2.2 Surgical Induction

Te surgical procedures used in this study for hydrocephalus rats model followed the procedures that are described in detail in our previous study [22]. The rats were anesthetized by i.p. injection of pentobarbital and were placed in a stereotaxic frame. The whole operation process followed the aseptic surgical techniques, and the tail vein was cannulated to facilitate the intravenous (i.v.) infusion of 10% HS or normal saline. The injection coordinates, which were measured from the bregma to the lateral cerebral ventricles, were 0.8 mm posterior, 1.6 mm lateral, and 3.7 mm deep. A 30‐μl sterile suspension of 3% kaolin (ultrasonic emulsification about 15 min) was injected slowly into the lateral cerebral ventricles (depth: 3.7 mm) at a rate of approximately 10 μL/min. The skin incision was sutured, and the animal was replaced in its cage and was monitored daily for the duration of the experiment.

### 2.3 Drugs Administration

**FIGURE 1:**
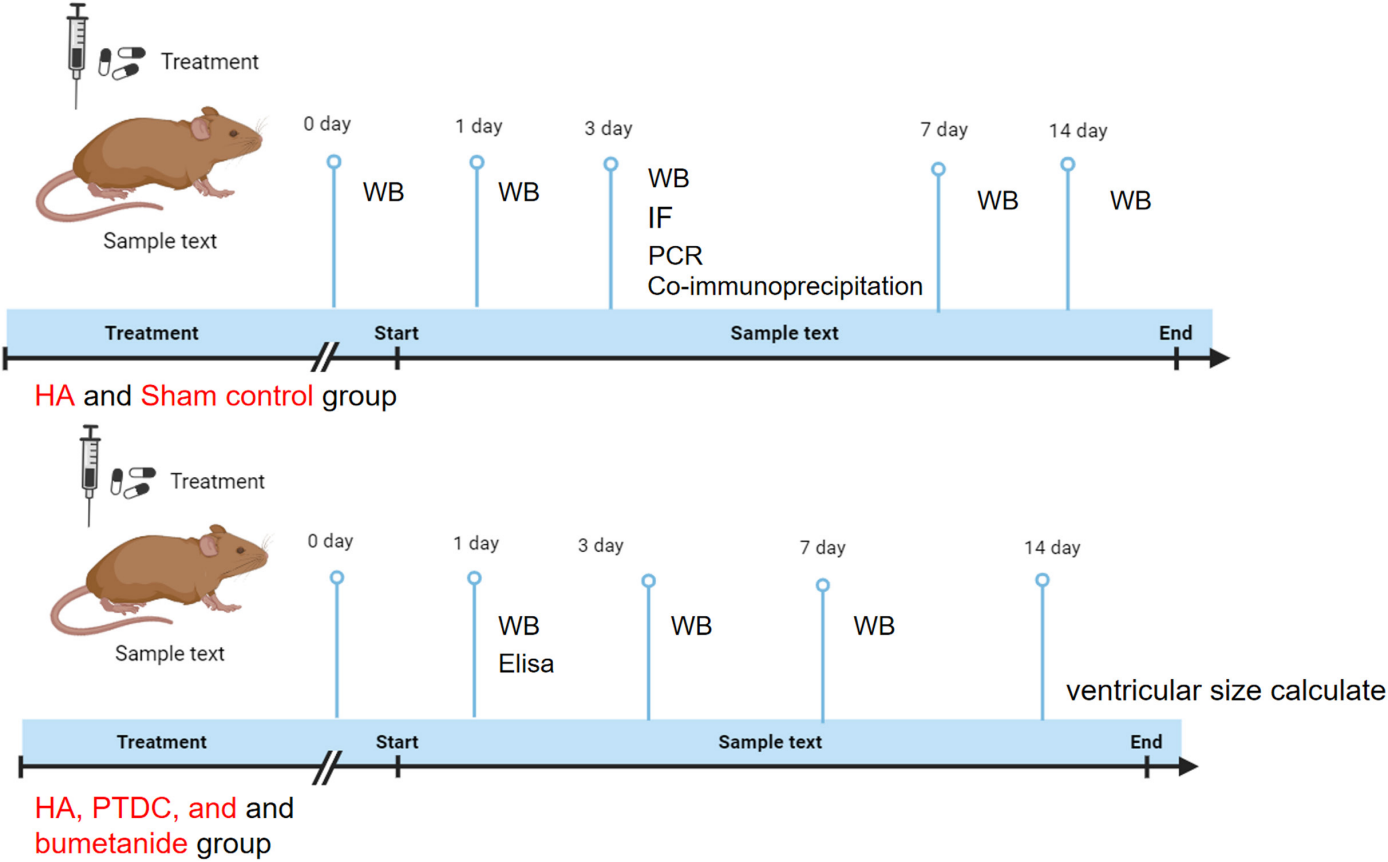
Schematic experimental design and subsequent analyses. HA, hydrocephalus model; IF, immunofluorescence; WB, Western blot.

After modeling, all rats were randomly divided into four groups as follows:Sham control (15 rats), HA (30 rats), HA + PTDC (hereafter referred to as PTDC group) (25 rats), and HA + bumetanide groups (hereafter referred to as bumetanide group)(25 rats). PDTC (1 mg/mL 25 mg in normal saline, i.p., Sigma‐Aldrich) and bumetanide (Bumetanide 0.25 mg/mL 30 mL, Sigma‐Aldrich) was administered i.p. from the time of operation and every 8 h thereafter until 36 h following operation in PDTC group and Bumetanide group. Rats in the sham group and hydrocephalus group were treated with i.v. infusion of normal saline instead. The Schematic experimental design and subsequent analyses were showed as Figure 1. Five experimental rats were sacrifice at each time point.

### 2.4 Sacrifice and Tissue Sample Collection

CSF samples can be collected from the lateral ventricle. These samples were centrifuged, then the supernatant fluids were collected and immediately stored at −20°C for further use in ELISA. Following i.p. injection of pentobarbital, the brain was promptly removed and dissected on ice. Samples from the periventricular tissue, parietal cortex (gray and white matter), and choroid plexus were was carefully dissected under magnification using sharp forceps and placed in sterile tubes and immediately submerged in liquid nitrogen, and then transferred and stored at −80°C until use. For histological analysis, rats were transcardially perfused with ice‐cold normal saline followed by 10% neutral‐buffered formalin. Brains were dissected and kept in formalin for 24 h before being transferred to a 30% sucrose solution for cryoprotection.

### 2.5 Western Blot

Brain tissues were collected and stored at −80°C after being perfused with cold PBS (0.1 M, pH 7.4) at 12 h, 1 day, 3 days, 5 days, 7 days for the time course study, and at 3 days for the mechanistic study after GMH induction, respectively. Western blot was performed as previously described [22]. After sample preparation, 50 μg protein per sample was loaded onto 10%–12% SDS‐PAGE gels, ran for 90 min at 100 V, and was transferred onto 0.2 mm or 0.45 mm nitrocellulose membranes at 100 V for 120 min (Bio‐Rad). The membranes were blocked for 2 h in 5% non‐fat milk in Tris‐buffered saline with 0.1% tween20, followed by overnight incubation at 4°C with the following primary antibodies (The antibodies used were showed in Table 2). Appropriate secondary antibodies (Santa Cruz, USA) were incubated with membranes for 2 h at room temperature. Bands were visualized using ECL Plus Chemiluminescence kit (Amersham Biosciences, USA) and quantified through ImageJ 4.0 (Media Cybernetics).

**TABLE 2:** Antibodies used in experiments.

Antibody	Host	Company	Cat. no.	Concentration
GFAP	Mouse	Millipore, Billerica, MA, USA	MAB360	1:100
AQP1	Mouse	Abcam, Cambridge, MA, USA	sc‐25287	1:200
NKCC1	Rabbit	Millipore, Billerica, MA, USA	AB3560P	1:100
pNKCC1	Rabbit	EMD Millipore, USA	ABS1004	1:200
pNA+/K + ATPnse	Rabbit	Cell Signaling Technology, Danvers, MA, USA	4006	1:200
TNF‐α	Rabbit	Millipore, Billerica, MA, USA	AB1837P	1:50
TLR4	Mouse	Abcam, Cambridge, MA, USA	sc‐293,072	1:100
Nf‐κb p65	Rabbit	Santa Cruz Biotechnology, Santa Cruz, CA, USA	sc‐372	1:200
SPEAK	Rabbit	Abcam, Cambridge, MA, USA	ab79045	1:200
pSPAK	Rabbit	EMD Millipore, USA	07–2273	1:200
GAPDH	Goat	Santa Cruz Biotechnology, Santa Cruz, CA, USA	sc‐20,357	1:1000
β‐Actin	Rabbit	Cell Signaling Technology, Danvers, MA, USA	4970	1:1000

### 2.6 Immunoblotting and Immunoprecipitation

Protein concentrations were determined by the BCA method [23] using the BCA‐100 protein quantitative analyzing kit (Shanghai Biocolor Bioscience & Technology Co. Ltd., Shanghai, China), and then the protein samples were heated at 95°C for 5 min and separated by SDS‐PAGE. The proteins were blotted onto polyvinylidene difluoride membrane and then blocked with 5% non‐fat milk for 1 h at room temperature. An overnight incubation with the primary antibodies followed the manufacturers recommendations. After rinses with Tris‐buffered saline with 0.1% Tween‐20, the membranes were incubated with the horseradish peroxidase (HRP)‐conjugated secondary antibodies for 1 h at room temperature. The primary antibodies for the tissue (Table 2) were as follows: TLR‐4, pNA+/K + ATPnse, pNKCC1, pSPAK, GFAP, AQP1 and AQP4. The secondary antibodies used were as follows: goat anti‐rabbit IgG‐HRP (Sanying, Wuhan, China, SA00013‐2), donkey anti‐goat IgG‐HRP (Sanying, Wuhan, China, SA00003‐3). The immunoblots were developed using the enhanced chemiluminescence detection system (Bei Jing Pu Li Lai Gene Technology Co. Ltd., China). Densitometrically analyzed using Image J, the freely available NIH image analysis software (http://rsbweb.nih.gov/ij/download.html).

SPAK and NKCC1 total antibodies and the phosphorylation‐site‐specific antibodies were coupled with Protein G–Sepharose at a ratio of 1 mg of antibody to 1 mL of beads in the presence of 20 μg/mL lysate to which the corresponding unphosphorylated peptide had been added. 200 μg of clarified cell lysate was incubated with 10 μg of antibody conjugated to 10 μL of Protein G–Sepharose for 2 h at 4°C with gentle agitation. Beads were washed three times with 1 mL of lysis buffer containing 0.15 M NaCl and twice with 1 mL of wash buffer (50 mM Tris/HCl, pH 7.5, and 0.1 mM EGTA). Bound proteins were eluted with 1 × LDS sample buffer (Invitrogen) containing 1% 2‐mercaptoethanol. IgG used in control immunoprecipitation experiments was affinity‐purified from preimmune serum using Protein G–Sepharose.

### 2.7 Elisa

The protein concentrations of IL‐6 and TNF‐α in each group of animals, were quantified using an ELISA kit (purchased from 4A Biotech Co. Ltd) according to the manufacturer's instruction. Finally, the reaction plates were read within 30 min in an enzyme micro‐plate reader at 450 nm.

### 2.8 Rt‐PCR

Total RNA was extracted by Trizol reagent (Invitrogen, USA) according to the manufacturer's instructions. The quantity of total RNA was measured with a UV Spectrophotometer (Biochrom Ltd., UK). For the reverse transcription, 2 μL of total RNA was combined with 4 μL of 5 × PrimeScript Buffer (PrimeScript RT Master Mix, TaKaRa Biotechnology (Dalian) Co. Ltd., China; Cat No: DRR036S). RNase Free dH2O was added to 20 μL, after which the mixture was heated at 37°C for 15 min, 85°C for 5 s. Quantitative RT‐PCR was carried out on a Light Cycler 480 instrument using a FastStart DNA Master plus SYBR Green I kit (Roche Diagnostics, GmbH, Roche Applied Science, Penzberg, Germany) according to the manufacturer's instructions. Reverse transcription and qRT‐PCR were performed using commercially available reagents (TaKaRa, Japan) with the StepOne Real‐Time PCR System (ABI, UK). Sybr Green I was used to detect amplification, and β‐actin was used as a normalizing control. Forward and reverse primers were as follows Table 3. The RT‐PCR consisted of two programs. The first one was complementary cDNA synthesis: one cycle at 50°C for 30 min and one cycle at 94°C for 2 min. The other one was second‐strand cDNA synthesis, and the PCR consisted of 45 cycles at 94°C for 30 s, 58°C for 30 s, and 72°C for 20s, followed by a final extension step at 72°C for 10 min. Calculations were performed by the CPR method. All samples were assayed in triplicates.

**TABLE 3:** Primer sequences applied for q‐PCR.

Primer	Forward prime 5′‐3′	Reverse prime 5′‐3′
NKCC1	AGACTTCAACTCAGCCACTGT	CAAGGTCAAACCTCCATCATCA
Nf‐κb p65	ACGATCTGTTTCCCCTCATC	GCCTGGGAAAGTCCCCTCAACT
IL‐18	GCAGTAACCATCTCTGTGCAGTGTA	TCATCAATATTATCAGGAGGACTACAT
TNF‐α	CCAACAAGGAGGAGAAGTTCC	CTCTGCTTGGTGGTTTGCTAC
β‐Actin	GATCAAGATCATTGCTCCTCCTG	AGGGTGTAAAACGCAGCTCA

### 2.9 Ventricular Volume Measure

We took high‐resolution pictures of serial coronal sections (200 μm apart, 14 levels) using uniform parameters of camera positioning, magnification, and external lighting. The volumetric calculations were semiautomated (by NIH Image) as follows: an appropriate intensity threshold was first chosen to exclude background tissue and to highlight the bright ventricles. This was followed by careful inspection of each image, and manual tracing was used to correct any areas of the ventricle which had been incorrectly deleted or to delete non‐ventricular regions that had been incorrectly included. This process resulted in a binary mask of ventricular pixels, which multiplied by the volume of each pixel and summed over all slices produced the net ventricular volume in milliliters.

### 2.10 Data Analysis

The data were analyzed and plotted by Graph PadPrism 7 and presented as the means±SEMs. Sample sizes were calculated using an a priori sample size calculator with the following assumptions: *A* = 0.05; two‐tailed; and desired power, 80%. The results indicated that a minimum of 3 rats per group were needed. All data were analyzed using GraphPad Prism and satisfed a normal distribution and the homogeneity of variance. The Shapiro–Wilk (SW) test was used to evaluate the normality of the data distributions. The normally distributed data were analyzed using Student's *t*‐test for two comparisons or one‐way ANOVA with Tukey's post hoc test for multiple comparisons. For non‐normally distributed data, the Mann–Whitney U test was employed as an alternative to the *t*‐test, and the Kruskal–Wallis test was selected as a replacement for ANOVA. A *p* value < 0.05 was considered to be statistically significant. The number of different experimental groups and statistical methods is shown in the figure legends, and statistical analysis data are detailed in the Appendix 1: Statistical results.

## 3 Results

### 3.1 AQP1 Activation and Inflammation in Hydrocephalus Rats Brain

**FIGURE 2:**
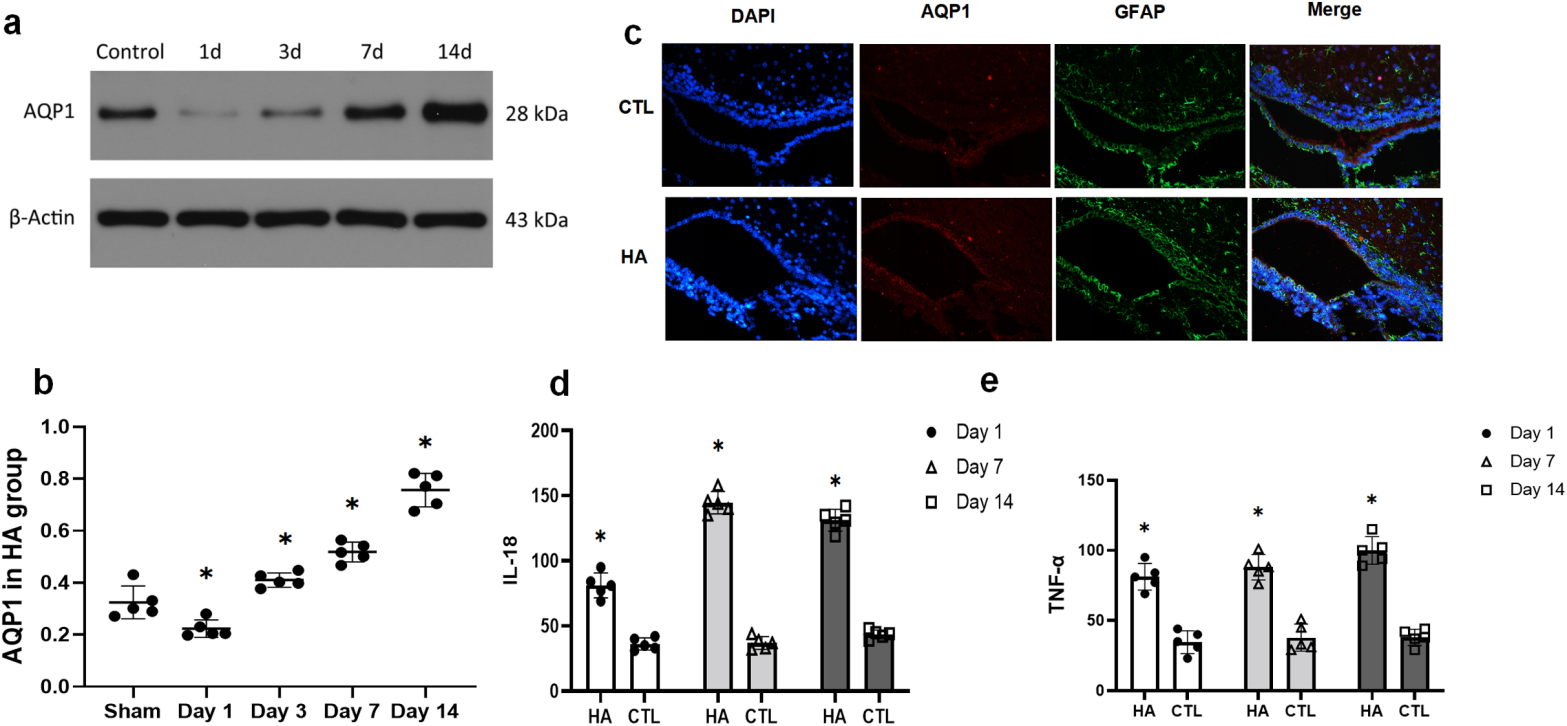
Inflammatory response and AQP1 expression (a and b). Representative protein expression levels of AQP1 and β‐Actin in the ventricular wall of the control, HA groups were evaluated. (c) Immunofluorescence staining of AQP1 in CPE. (d and e). Elisa verified the expression changes of IL‐18 and TNF‐α in cerebrospinal fluid in hydrocephalus group and control group (*p < 0.05 in comparison with the control group).

To detect the CPE inflammation, GFAP and APQ1 immunofluorescence were performed double staining. It can be found that the expression trend was similar. According to the WB result, AQP 1 shortly decreased in 1 day post‐operation (*p* = 0.0135, *t* = 3.156). Then it continued to increase and significantly high expression from the 3 days (*p* = 0.0238, *t* = 2.784) to 14 days post‐operation (*p* < 0.0001, *t* = 10.68)(Figure 2a,b). The inflammation process was also proved by the TNF‐α and IL‐18 expression in 3 days post‐operation (Figure 1c,d).

### 3.2 NKCC1 Expression and TLR4/NF‐κB/NKCC1 Activation CPE

**FIGURE 3:**
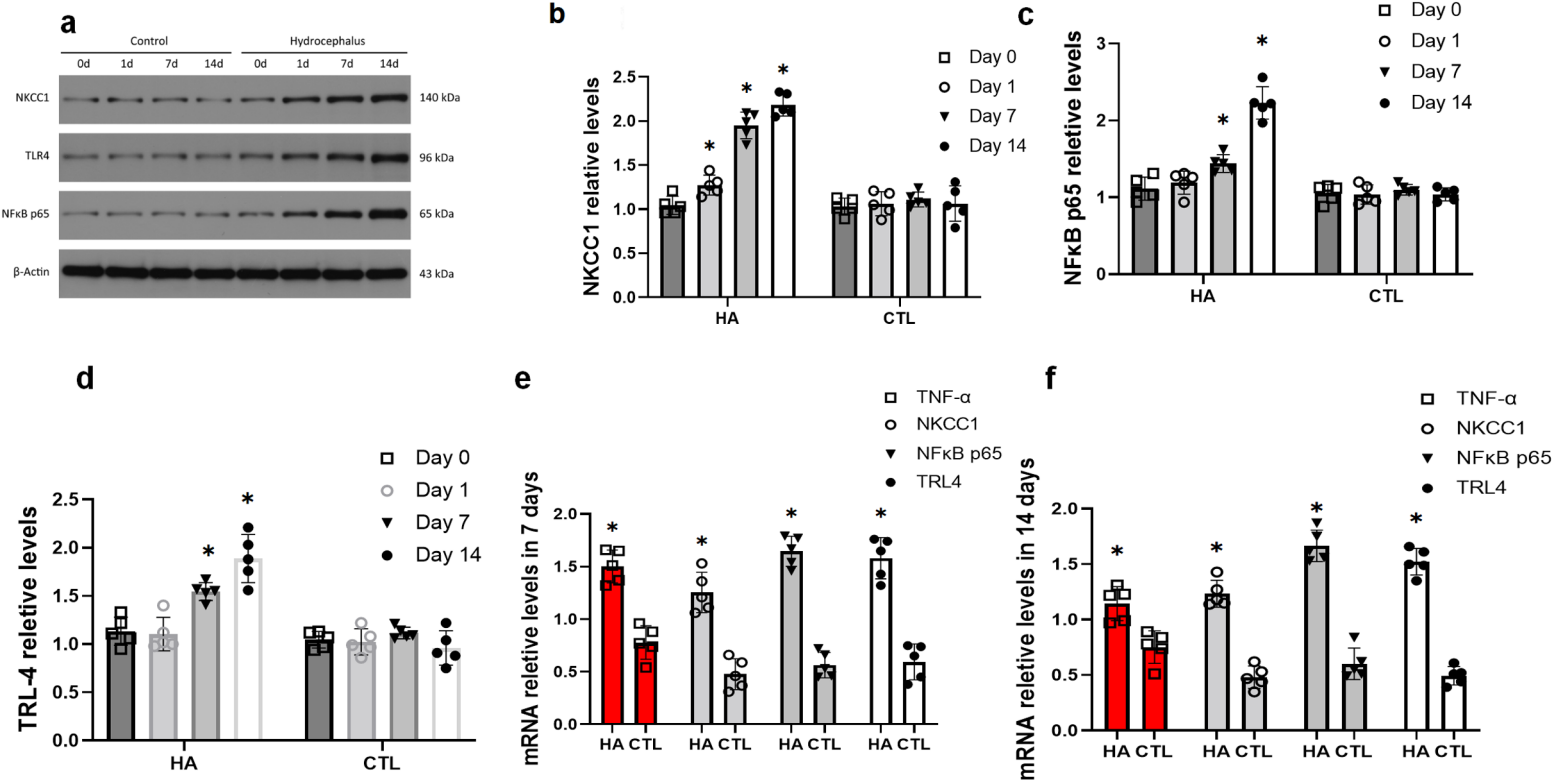
Inflammatory response and expression of TNF‐α, NKCC1, TLR4 and NF‐κB p65 WB in choroid plexus of hydrocephalus control group. (a) WB staining bands of NKCC1, TLR4 and NF‐κB P65. (b) Representative protein expression levels of NKCC1 showed an increasing trend from 1 to 14 days after operation. (c) Representative protein expression levels of TLR4 showed an increasing trend from 1 to 14 days after operation; (d) Representative protein expression levels of TLR4 showed an increasing trend from 1 to 14 days after operation; (e and f) RT‐PCR was used to verify the mRNA expression of NKCC1, TLR4, NF‐κB p65 and TNF‐α in hydrocephalus group and control group (*p < 0.05 in comparison with the control group).

To determine the profile of NKCC1, we analyzed the protein and mRNA expression of NKCC1 at 1 day following the operation. Our results showed an upregulation of the protein and mRNA expression of NKCC1 in the HA CPE in 1 to 7 days (*p* < 0.05) (Figure 3a,b). The optical density of the immune‐reactive bands of NKCC1 protein levels significantly increased 7 to 14 days compared with that in the control group (*p* < 0.05) (Figure 3c,d).

We also detected molecular NKCC1, TLR4 and NF‐κB p65. Our results showed increased expression of NKCC1, TLR4 and NF‐κB in HA group as compared to Sham controls at 1d, 7d, 14d post‐operation. To determine the inflammation condition, we detected the IL‐18, TNF‐α concentrations level in CSF. The TNF‐α and IL‐18 concentrations were significantly higher in the model group than in controls in 7 and 14 days (*p* < 0.05) (Figure 2e,f). Following previous reports [6, 15], these results demonstrate that Kaolin‐injection triggers CPE inflammation, dependent on NF‐κB activation.

### 3.3 NKCC1‐SPAK Complex Activation

**FIGURE 4:**
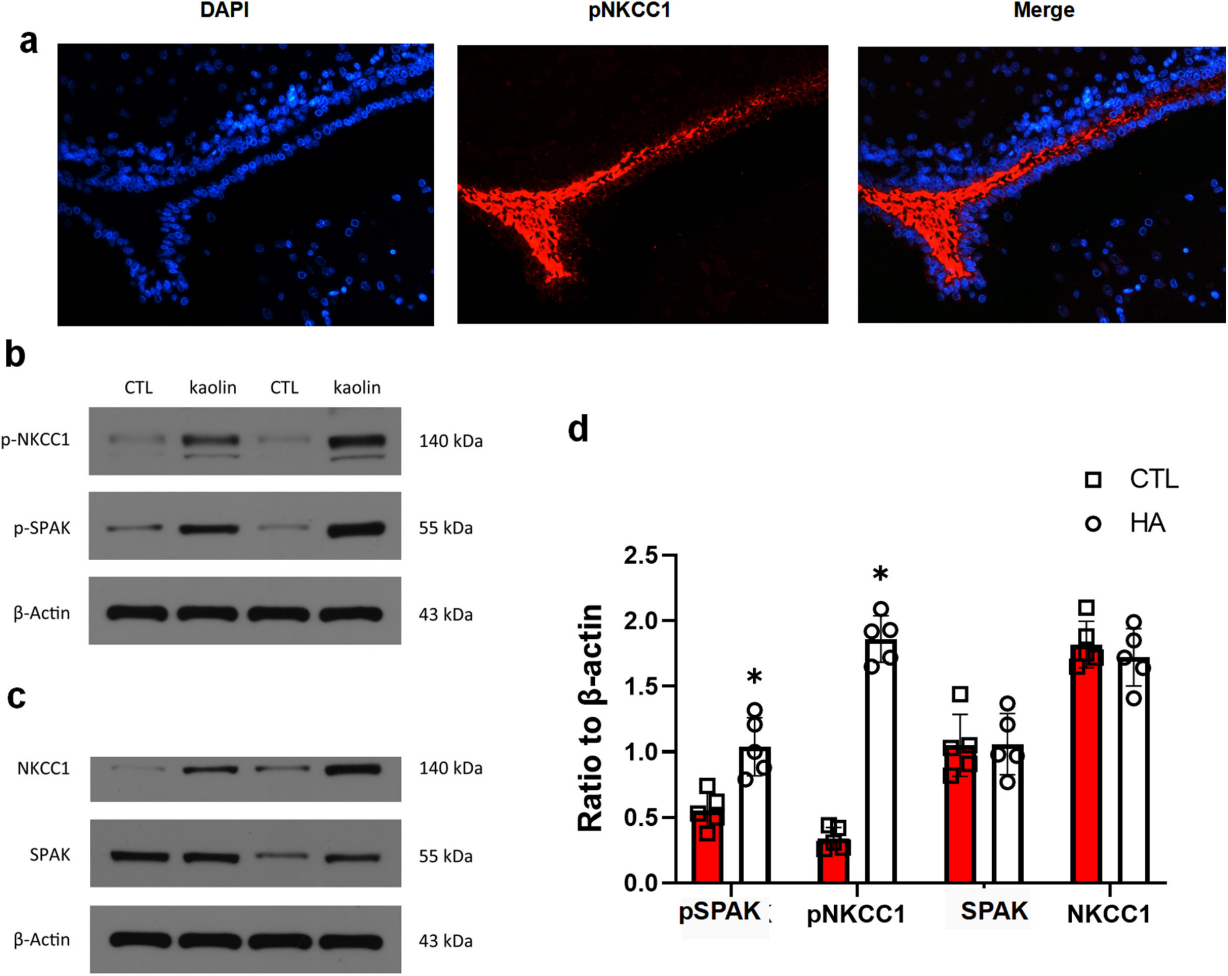
The immunofluorescence and co‐immunoprecipitation pNKCC1‐pSPAK complex. (a) immunofluorescence of pNKCC1 (b) WB staining bands of NKCC1 and SPAK. (c) WB staining bands of pNKCC1 and pSPAK. (d) Representative protein expression levels of NKCC1, SPAK, pNKCC1 and pSPAK (*p < 0.05 in comparison with the control group).

According to our immunofluorescence result, pNKCC1 had high expression in CPE in 3 days post‐operation. In CPE, SPAK and NKCC1 are in a complex form called NKCC1‐SPAK complex, which can be decteded by reciprocal coimmunoprecipitation experiments with CPE homogenates. This complex included the phosphorylated, active species of both SPAK and NKCC1. The interaction between pSPAK (*p* = 0.0031, *t* = 4.185) and pNKCC1 (*p* < 0.0001, *t* = 17.36) was increased in the CPE of the HA group compared with the sham control group in 3 days, while the SPAK (*p* = 0.948, *t* = 0.06725) and NKCC1 (*p* = 0.4595, *t* = 0.7771) showed no significant difference (*p* > 0.05) (Figure 4).

### 3.4 Effects of Regulation TLR4/NF‐κB/NKCC1 on Neuro‐Inflammation and Ventricle Enlargement

**FIGURE 5:**
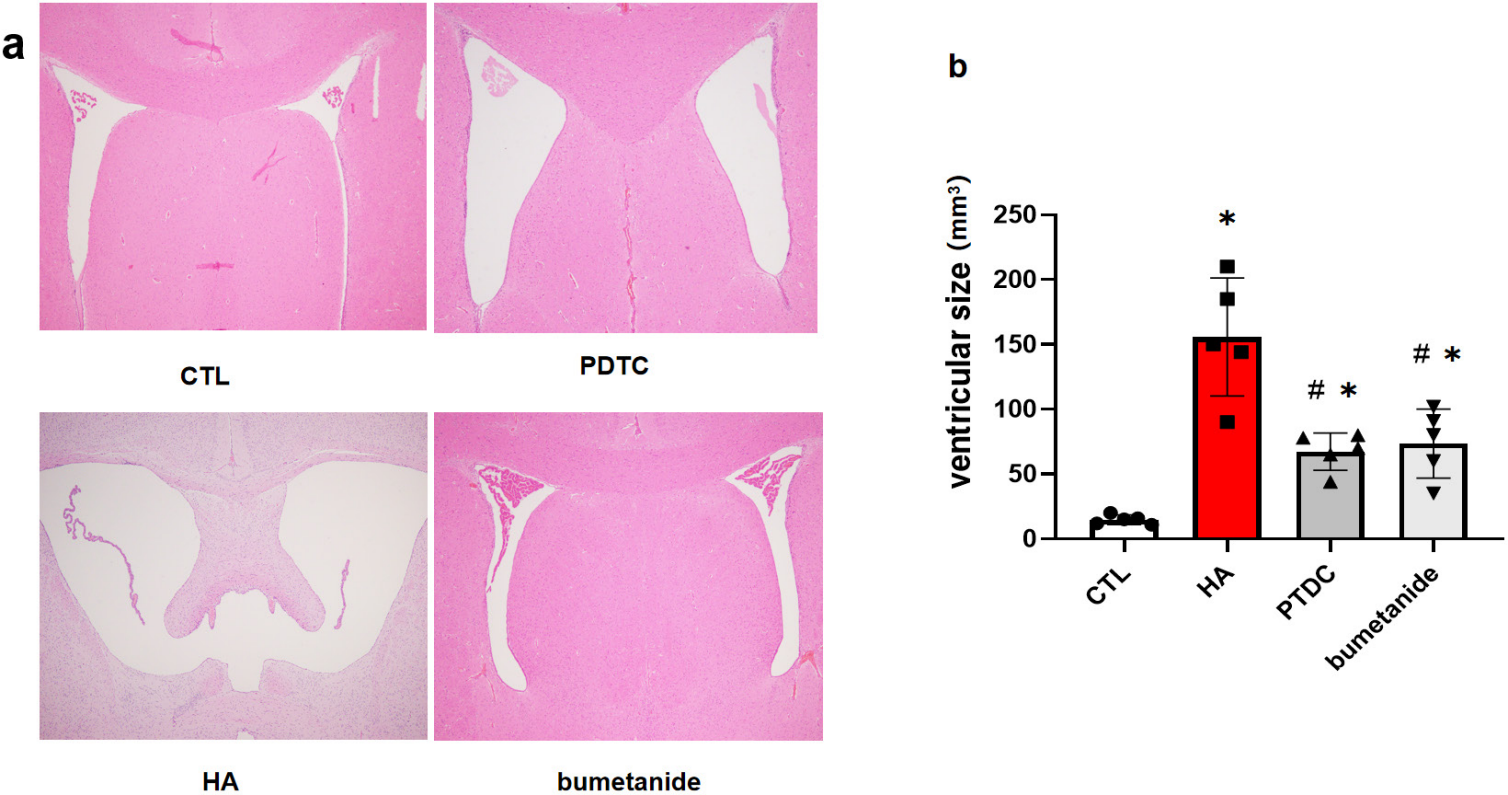
(a) Representative photomicrographs of coronal sections of brains in 14 days post operation. (b) the ventricular size in control, PDTC, bumetanide and HA groups (*p < 0.05 in comparison with the control group; #*p* < 0.05 in comparison with the HA group).

We next evaluated the effect of neuroinflammation and ventricle enlargement by regulating TLR4/NF‐κB/NKCC1. We used NF‐κB inhibitor PDTC and NKCC1 cotransporter inhibitor bumetanide. In both PDTC and bumetanide groups, the ventricle enlargement was alleviated in 14 days post‐operation compared to the HA group (*p* < 0.05) (Figure 5).

**FIGURE 6:**
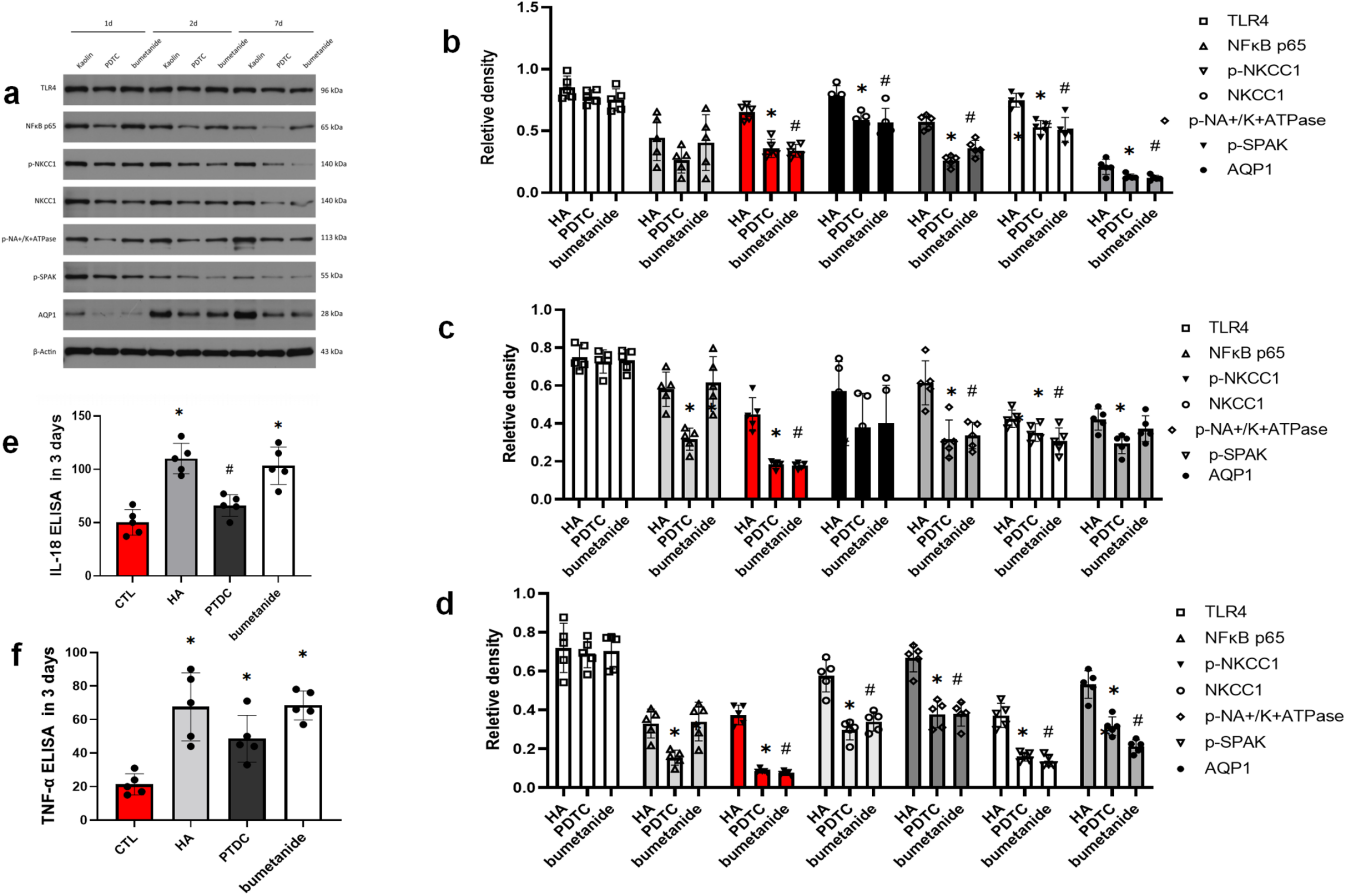
Expression of TLR4, p65, pNKCC1, pSPAKand AQP1 in the CPE of PDTC and bumetanide treatment. (a–c) WB staining bands of TLR4, p65, pNKCC1, pSPAK and AQP1. (d) Representative protein expression levels of TLR4, p65, pNKCC1, pSPAK and AQP1 in 1 day, 3 days and 7 days respectively postoperation. (e and f) Elisa verified the expression changes of IL‐18 and TNF‐α in cerebrospinal fluid in hydrocephalus group and control group in 7 days postoperation (*p < 0.05 in comparison with the control group; #*p* < 0.05 in comparison with the HA group).

We detected that the TLR4, p65, pNKCC1, pSPAK and AQP1 presented an upward trend in 1–3 days post‐surgery. Bumetanide is an NKCC1 cotransporter inhibitor, reduced the pNKCC1 and also down regular the expression of AQP1. PDTC reduced NF‐κB expression and led to the p65, pNKCC1. Through anti‐inflammatory effects. AQP1 expression decreased 3 days postoperatively, possibly reducing CSF secretion. In the bumetanide group, the IL‐18 and TNF‐α were not impacted according to the ELISA results (*p* < 0.05). In the PDTC group, the inflammation level was also alleviated by IL‐18 and TNF‐αreduced in 3 days post‐operation (*p* < 0.05) (Figure 6).

## 4 Discussion

Hydrocephalus is actually a metabolic imbalance of fluid within the brain. AQP is a recently discovered protein that regulates fluid homeostasis in various organs, and several subtypes have been reported [22]. AQP 1 is abundant in the choroid plexus, regulating CSF physiology. It can transport water bi‐directionally across cell membranes in response to changes in passive osmotic pressure gradients as they have a high capacity and more excellent selectivity for the water molecules. The selectivity of these channels allows for water to pass through but not acid. AQPs have a narrow pathway that is a very tight fit for water, and the mechanism in the pore allows water molecules to pass through in a single file with no resistance [24, 25]. This mechanism allows AQPs to transport water across cell membranes rapidly. However, several proteins such as NKCC1 and GLUT1 expressed at the endothelium co‐transport water independently of osmotic gradients along with their substrates [26].

**FIGURE 7:**
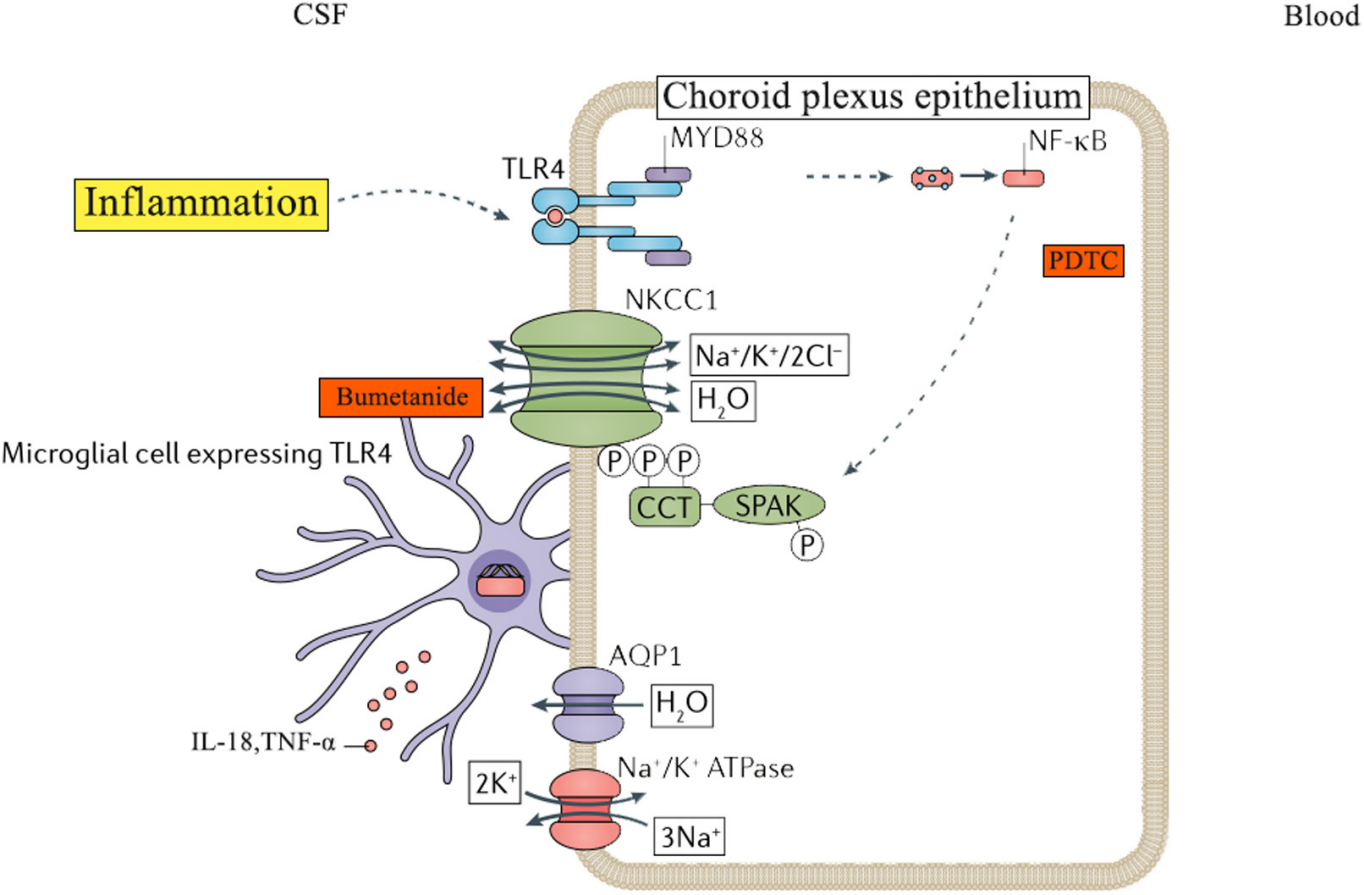
TLR4/NF‐κB/NKCC1 and proposed mechanism of CSF hypersecretion.

The stability of CSF homeostasis is extremely fragile. When pathogenic molecules enter the CSF system, such as methemoglobin and lipopolysaccharide, whatever there are Host‐derived or pathogen‐associated, TLR4 on the surface of the CPE could be activated [27]. This binding stimulates a TLR4‐MYD88 signaling cascade leading to NF‐κB. Nuclear NF‐κB stimulates the production of proinflammatory cytokines, for example, tumor necrosis factor‐α (TNFα) and IL‐1β, which increase the activity of STE20/SPS1‐related proline/alanine‐rich kinase (SPAK). SPAK phosphorylates its canonical substrate, the Na+/K+/2Cl ion cotransporter (NKCC1). NKCC1 phosphorylation increases the activity of the transporter, which results in a net increase in CSF production by the CPE [28]. At the same time, an inflammatory response is occurring in the cerebrospinal fluid, and proinflammatory factors are released from the surface of the glial cells [29]. These cytokines can also bind receptors on the CPE and propagate CPE inflammation and CSF hypersecretion. Aquaporin 1 (AQP1), anion exchange protein 2 (AE2), and sodium‐driven chloride bicarbonate exchanger (NCBE) are transporter proteins that facilitate the passage of water (AQP1) and ions (AE2 and NCBE) across the plasma membrane (Figure 7) [1].

In this study, a kaolin hydrocephalus model was selected to investigate the relationship between cerebrospinal fluid choroid plexus inflammatory response and the development of hydrocephalus. The Kaolin‐induced hydrocephalus model is the most commonly used one for the experimental research of the disease [20, 30]. Kaolin deposition in subarachnoid space can also lead to inflammation in the cerebrospinal fluid circulation system and elevation of many proinflammatory factors [23]. The ability to stably and specifically target AQP is suitable intervention drugs. Some studies have demonstrated that bumetanide, an NKCC1 inhibitor, reduces CSF secretion at the choroid plexus epithelium [5, 31]. Recently, further evidence has emerged to support the involvement of NKCC1 in CSF secretion independently of osmotic driving forces. NKCC1 contributes to approximately half of the CSF production by cotransport of water and its directional translocation of ions independently of an osmotic gradient [32].

TLR4 is a critical target in this study. TLR4 inhibition decreases inflammatory injury and neurological deficits after intraparenchymal hemorrhage [32], and TLR4 and its downstream pathway components (NF‐κB and the NF‐κB‐dependent cytokines TNF‐α and IL‐1β) are upregulated in the CPE. Furthermore, at physiological concentrations, intracerebroventricular delivery of metHgb activates TLR4 homodimers or TLR4/2 heterodimers, promoting nuclear translocation of NF‐κB as well as secretion of TNFα and IL‐1β [33, 34, 35], and is sufficient to cause ventriculomegaly [5, 34]. In addition, in a rat model of post‐hemorrhagic hydrocephalus, NKCC1 was hyper‐activated by inflammatory markers in the CSF and caused bumetanide‐sensitive ventriculomegaly [36]. One study proposes that some of the inhibitory actions of bumetanide, a drug thought to act on NKCC1 channels, occur through inhibiting AQP1 at high concentrations [37, 38, 39, 40].

This study also indicated that CSF hypersecretion mediated by the SPAK‐NKCC1 complex is dependent on TLR4‐NF‐κB signaling. SPAK is a signal transducer of environmental and cellular stress signals, including NF‐κB‐dependent inflammationInterferon (IFN)‐γand TNF‐αstimulate SPAK signaling in an NF‐κB‐dependent manner to increase epithelial transport in experimental colitis. SPAK could therefore be well‐positioned to mediate an epithelial pro‐secretory response to inflammation dependent on TLR4–NF‐κB signaling. However, CSF hypersecretion from the CPE may resemble the hypersecretory phenotypes in other inflamed epithelia [41, 42].

Previous studies have shown that the inflammatory response primarily affects CSF absorption. However, a series of recent studies have shown that the inflammatory response of CSF, due to the early increase of CSF secretion, leads to a series of disorders of CSF metabolism, which is similar to many other secretory diseases. It will lead to structural deformation of the ventricle and subsequent impaired absorption of the entire CSF circulation. The secretion and absorption of CSF fluid do not act independently but are related. In particular, AQP 1 is abundant in the choroid plexus, where it regulates CSF physiology, and AQP 4 is distributed in the brain parenchyma. They interact and regulate water physiology [33, 43, 44, 45, 46].

This study further validates the hypothesis of previous research that CPE secreted higher volumes of CSF and depended on the expression of AQP1 and Na+, K+, Cl−, and water cotransporter NKCC1. In addition, it also showed that, under inflammatory conditions, TLR4‐dependent activation, Ste20‐type stress kinase SPAK and pNKCC cotransporter expression boosting, and NKCC1 binding and phosphorylation in choroid plexus epithelial cells enhanced. It will result in the AQP1 activation abnormal secretion of CSF. Interventions in the early stage of hydrocephalus (within 3 days) may reduce the abnormal secretion of cerebrospinal fluid and the occurrence of hydrocephalus by inhibiting the activation of TLR4/NF‐κB/NKCC1 pathway.

## 5 Conclusion

These results further our understanding of the pathogenesis of hydrocephalus and may lead to new treatment strategies by inhibiting TLR4‐NF‐κB to modulate inflammation in the hydrocephalic brain, thereby, brain function damage can be mitigated, and even the shunt related hydrocephalus process can be avoided.

## Author Contributions

Hao Xu and Jiawei He worked on the experimental design. Hao Xu and Jiawei He conducted the experiments, analyzed the data, and drafted the manuscript. Xinfeng Liu worked on the manuscript revision. Hua Du and Xiaolei Jin participated in the experimental design, data analysis and interpretation, and manuscript preparation. The authors read and approved the final manuscript.

## Ethics Statement

Humans were not used in this study. All animal experiments were approved by the USTC Animal Care and Use Committee.

## Consent

The authors have nothing to report.

## Conflicts of Interest

The authors declare no conflicts of interest.

## Data Availability

The authors confirm that all data underlying the findings are fully available without restriction. Any additional information can be addressed via contact with the corresponding author of this manuscript.

## Appendix 1

### Figure 2: Descriptions

(b) Aqp1 in HA group: *p* = 0.0002, F=24.21. Day 1 vs. Day 0: *p* = 0.0135, *t* = 3.156; Day 3 vs. Day 0: *p* = 0.0238, *t* = 2.784; Day 7 vs. Day 0: *p* = 0.0.004, *t* = 5.882; Day 14 vs. Day 0: *p* < 0.0001, *t* = 10.68;

(c) IL‐18 HA vs. CTL in 1 day: *p* < 0.0001, *t* = 9.470; HA vs. CTL in 7 days: *p* < 0.0001, *t* = 24.39; HA vs. CTL in 14 days: *p* < 0.0001, *t* = 21.57.

(d) TNF‐αHA vs. CTL in 1 day: *p* < 0.0001, *t* = 8.303; HA vs. CTL in 7 days: *p* < 0.0001, *t* = 8.493; HA vs. CTL in 14 days: *p* < 0.0001, *t* = 12.16.

### Figure 3: Descriptions

(b) HA vs. CTL in 0 day: *p* = 0.8292, *t* = 0.2229; HA vs. CTL in 1 day: *p* = 0.0279, *t* = 2.679; HA vs. CTL in 7 days: *p* < 0.0001, *t* = 10.90; HA vs. CTL in 14 days: *p* < 0.0001, *t* = 10.52.

(c) HA vs. CTL in 0 day: *p* = 0.4976, *t* = 0.7105; HA vs. CTL in 1 day: *p* = 0.1167, *t* = 1.759; HA vs. CTL in 7 days: *p* = 0.0005, *t* = 5.682; HA vs. CTL in 14 days: *p* < 0.0001, *t* = 11.67.4.

(d) HA vs. CTL in 0 day: *p* = 0.2809, *t* = 1.156; HA vs. CTL in 1 day: *p* = 0.4397, *t* = 0.8130; HA vs. CTL in 7 days: *p* < 0.0001, *t* = 8.666; HA vs. CTL in 14 days: *p* = 0.0001, *t* = 6.778.

(e) NKCC1 HA vs. CTL in 1 day: *p* < 0.0001, *t* = 7.255; TLR4 HA vs. CTL in 7 days: *p* < 0.0001, *t* = 7.180; NF‐κB p65 HA vs. CTL in 14 days: *p* < 0.0001, *t* = 12.88; TNF‐α p65 HA vs. CTL in 14 days: *p* < 0.0001, *t* = 8.513.

(f) NKCC1 HA vs. CTL in 1 day: *p* = 0.0033, *t* = 4.121; TLR4 HA vs. CTL in 7 days: *p* < 0.0001, *t* = 10.68; NF‐κB p65 HA vs. CTL in 14 days: *p* < 0.0001, *t* = 11.85; TNF‐α p65 HA vs. CTL in 14 days: *p* < 0.0001, *t* = 15.68.

### Figure 4: Descriptions

(d) pSPAK HA vs. CTL: *p* = 0.0031, *t* = 4.185; pNKCC1 HA vs. CTL: *p* < 0.0001, *t* = 17.36; SPAK HA vs. CTL: *p* = 0.948, *t* = 0.06725; NKCC1 HA vs. CTL: *p* = 0.4595, *t* = 0.7771.

### Figure 5: Description

(b) HA vs. CTL *p* = 0.0001, *t* = 6.903; PTDC vs. CTL *p* < 0.0001, *t* = 7.921; bumetanide vs. CTL *p* = 0.0012, *t* = 4.903;PTDC vs. HA *p* = 0.0033, *t* = 4.139;bumetanide vs. HA *p* = 0.0082, *t* = 3.486.

### Figure 6: Descriptions

(b) TLR4 HA vs. PDTC *p* = 0.1643, *t* = 1.531; HA vs. Bumetanide *p* = 0.2546, *t* = 1.227; NFκB p65 HA vs. PDTC *p* = 0.3679, *t* = 0.9542; HA vs. Bumetanide *p* = 0.4355, *t* = 0.8209; p‐NKCC1 HA vs. PDTC *p* = 0.0001, *t* = 6.831; HA vs. Bumetanide *p* < 0.0001, *t* = 8.701; NKCC1 HA vs. PDTC *p* = 0.0031, *t* = 4.175; HA vs. Bumetanide *p* = 0.0006, *t* = 5.470; p‐NA+/K+ ATPase HA vs. PDTC *p* = 0.0003, *t* = 6.185; HA vs. Bumetanide *p* = 0.0006, *t* = 5.470 p‐SPAK HA vs. PDTC *p* < 0.0001, *t* = 17.36; HA vs. Bumetanide *p* = 0.0016, *t* = 4.660; AQP1 HA vs. PDTC *p* = 0.028, *t* = 2.679; HA vs. Bumetanide *p* = 0.0162, *t* = 3.035.

(c) TLR4 HA vs. PDTC *p* = 0.5953, *t* = 0.5532; HA vs. Bumetanide *p* = 0.6937, *t* = 0.4083; NFκB p65 HA vs. PDTC *p* = 0.0006, *t* = 5.427; HA vs. Bumetanide *p* = 0.6347, *t* = 0.4938; p‐NKCC1 HA vs. PDTC *p* = 0.0002, *t* = 6.582; HA vs. Bumetanide *p* = 0.0001, *t* = 6.831; NKCC1 HA vs. PDTC *p* = 0.1099, *t* = 1.798; HA vs. Bumetanide *p* = 0.1744, *t* = 1.491; p‐NA+/K+ ATPase HA vs. PDTC *p* = 0.0025, *t* = 4.324; HA vs. Bumetanide *p* = 0.0017, *t* = 5.470; p‐SPAK HA vs. PDTC *p* = 0.0358, *t* = 2.520; HA vs. Bumetanide *p* = 0.0131, *t* = 3.176; AQP1 HA vs. PDTC *p* = 0.0057, *t* = 3.746; HA vs. Bumetanide *p* = 0.257, *t* = 1.220.

(d) TLR4 HA vs. PDTC *p* = 0.4232, *t* = 1.531; HA vs. Bumetanide *p* = 0.2546, *t* = 0.2466; NFκB p65 HA vs. PDTC *p* = 0.0016, *t* = 4.685; HA vs. Bumetanide *p* = 0.8639, *t* = 0.1771; *p*‐NKCC1 HA vs. PDTC *p* < 0.0001, *t* = 12.56; HA vs. Bumetanide *p* < 0.0001, *t* = 12.98; NKCC1 HA vs. PDTC *p* = 0.0002, *t* = 6.392 HA vs. Bumetanide *p* = 0.0006, *t* = 5.409; *p*‐NA+/K+ ATPase HA vs. PDTC *p* = 0.0002, *t* = 6.547; HA vs. Bumetanide *p* = 0.0001, *t* = 6.933; p‐SPAK HA vs. PDTC *p* < 0.0001, *t* = 7.156; HA vs. Bumetanide *p* < 0.0001, *t* = 7.684; AQP1 HA vs. PDTC *p* = 0.0005, *t* = 0.0005; HA vs. Bumetanide *p* < 0.0001, *t* = 9.115.

(e) HA vs. CTL *p* = <0.0001, *t* = 7.156; PTDC vs. CTL *p* = 0.0548, *t* = 2.247; bumetanide vs. CTL *p* = 0.0005, *t* = 5.589; PTDC vs. HA *p* = 0.0005, *t* = 5.597; bumetanide vs. HA *p* = 0.5336, *t* = 0.6504.

(f) HA vs. CTL *p* = 0.0012, *t* = 4.864; PTDC vs. CTL *p* = 0.0042, *t* = 3.962; bumetanide vs. CTL *p* < 0.0001, *t* = 9.817;PTDC vs. HA *p* = 0.1227, *t* = 1.726;bumetanide vs. HA *p* = 0.9373, *t* = 0.08122.
